# Goniowash: a new surgical approach combined with cataract surgery to lower intraocular pressure in pseudoexfoliation syndrome

**DOI:** 10.1007/s10792-020-01459-5

**Published:** 2020-06-26

**Authors:** Tao V. Tran, Kaweh Mansouri, Andre Mermoud

**Affiliations:** 1 Center for Ophthalmic Specialized Care (COS), Clinic Montchoisi, Ave Beaumont 9, 1012 Lausanne, Switzerland; 2 Glaucoma Research Center, Montchoisi Clinic, Swiss Visio, Lausanne, Switzerland; 3 Department of Ophthalmology, University of Colorado, Denver, USA

**Keywords:** Goniowash, Irrigation cannula, Pseudoexfoliation, Cataract, Glaucoma

## Abstract

**Purpose:**

Pseudoexfoliation syndrome is common in elderly patients and is associated with intraocular pressure elevation. Goniowash is a novel surgical technique to remove pseudoexfoliation material from the irido-corneal angle to decrease intraocular pressure. We assessed the long-term efficacy of Goniowash on relevant parameters in patients with pseudoexfoliation syndrome, after cataract surgery.

**Methods:**

The study enrolled 122 patients with pseudoexfoliation syndrome, who underwent routine cataract surgery combined with Goniowash. Best-corrected visual acuity, intraocular pressure and medication status were recorded in study participants during 5 years of follow-up.

**Results:**

Data from 190 eyes (122 patients of average age 73.8 ± 7.7 years) were assessed. Mean best-corrected visual acuity increased from 0.60 to 1.0 (*p* < 0.001) one year after surgery and remained stable throughout the follow-up. Average intraocular pressure decreased from 26.4 ± 7.3 mmHg pre-operatively to 15.9 ± 3.0 mmHg post operatively at one year (*p* < 0.001), 16.1 ± 3.3 mmHg (*p* < 0.001) at 3 years and 16.8 ± 2.9 (*p* < 0.001) at 5 years. Mean number of ocular hypotensive medications decreased from 1.5 ± 0.8 pre-operatively to 0.4 ± 0.7 post-operatively (75% reduction) (*p* < 0.001). No unexpected and severe adverse events related to the surgical procedure were reported.

**Conclusions:**

Goniowash combined with cataract surgery provides stable and long-lasting reduction of intraocular pressure and hypotensive medications. It is a safe procedure and may be an alternative for patients with pseudoexfoliation syndrome and elevated intraocular pressure.

## Introduction

Pseudoexfoliation syndrome (PEXS) is a systemic disease characterized by the progressive formation and accumulation of fibrillary deposits in various tissues and organs [1–3]*.* Ocular involvement in this syndrome is manifested by the chronic accumulation of an abnormal fibrillar matrix product or a complex of glycoproteins/proteoglycans [4] on the ciliary body, the zonules [5], the anterior surface of the lens [6], the iris, the edge of the pupil, the corneal endothelium, in the irido-corneal angle (ICA), on the trabecular meshwork (TM) and in Schlemm’s canal [3, 7–9]. Although the pathophysiology of this phenomenon is unclear, this anatomical distribution would support the hypothesis by which the origin of these microparticles originate from the level of the ciliary body [10].

The pseudoexfoliation (PEX) particles are insoluble and follow the natural flow of aqueous humor to be finally deposited within the TM. These deposits slowly impede the physiological outflow of the aqueous humor and lead to intraocular pressure (IOP) elevation. In addition, this type of glaucoma is characterized by high-pressure fluctuations and responds incompletely and inconsistently to topical drug treatments. It exposes patients to an accelerated optic neuropathy progression and perimetric impairment. On clinical examination, gonioscopy reveals PEX deposits in the TM. Examination by ultrasound biomicroscopy (UBM) can objectify these particles in the ICA, which are denser in the lower half [11]. Exfoliation material deposits contain lysosomal enzymes that lead to degeneration and dysfunction of the structures involved in the filtration process and these modifications are responsible for some pathological changes in the anterior segment such as zonular weakness, iridopathy, blood-aqueous barrier breakdown, ciliary body involvement, trabecular impairment and keratopathy [12].

Phacoemulsification is considered to be safe in most eyes with pseudoexfoliation even though significantly more complications occur intraoperatively. Glaucoma surgery is frequently indicated in the presence of PEX accompanied by ocular hypertension or exfoliative glaucoma (XFG). Trabeculectomy, non-penetrating glaucoma surgery, trabecular aspiration or phacoemulsification are valid treatment options for XFG. Each of them, however, is associated with frequent complications, regardless of the chosen technique [13–16]. Nevertheless, as of today, trabeculectomy with an adjunctive antimetabolite is considered as the gold standard surgical approach for the treatment of XFG [17].

This study presents a new method, termed “Goniowash”, combining cataract surgery with a simple and additional washout procedure of the TM and the ICA, performed in patients with PEXS and cataract. This technique was designed to eliminate the exfoliative material located on the TM and in the ICA in order to restore the physiological pathway of aqueous humor. The design concept of using pressure irrigation to clean the PEX material via a special cannula (Grieshaber Cannula, Grieshaber, Switzerland) was developed in our centre 12 years ago. Between 2007 and 2014, it was used in over 100 patients and a positive impact on IOP values and post-operative glaucoma medications was noticed. The purpose of this retrospective study was to evaluate the long-term safety and efficacy of the device in patients undergoing combined cataract surgery and Goniowash.

## Materials and methods

The study was formally approved by the Swissethics (approval #2017-00462). All study participants, prior to enrolment, signed an informed consent form, prepared in accordance with principles stated in the Declaration of Helsinki. All data collected within the scope of the study was anonymized, in order to guarantee protection of patients’ privacy. Each patient enrolled in the study was treated at Montchoisi Clinic, Lausanne by experienced ophthalmic surgeons.

The surgical procedure was performed following previously described approach [11]. The procedure started with cataract surgery performed according to each surgeon’s routine technique. When deemed necessary, it was complemented by other procedures, such as staining of the capsule (in case of important opalescence of the cataract) or the use of pupil dilatation (in case of a small pupil) [18]. Once the introduction of the intraocular lens (IOL) into the capsular bag was accomplished, wash out of the pseudo-exfoliative particles followed. To achieve this step, an irrigation cannula capable of irrigating the ICA and TM was used. Unlike a conventional single jet irrigation cannula, this new instrument is characterized by two distinct water jet modes, separated from each other by a 30° angle. With the special design of this cannula and micro-hydrodynamic laws, these two independent jets can be merged into a single, stronger jet. The two jets unify, with their power depending on the height of the bottle of BSS, located usually at the height of 80 to 90 cm over the patient’s eye. The cannula was used for subsequent irrigation of the TM and the ICA, in order to remove the PEX material and pigment accumulation. Due to a greater accumulation of material in the lower quadrants, the washout time for the lower area was usually longer, lasting about two minutes in the 3 and 9 o’clock segments, and about one minute for the upper area. Following a 360° irrigation of the ICA, the irrigation cannula is used to clean other parts of the anterior chamber, in particular, the edge of the pupil, the anterior surface of the iris, and optionally, surfaces behind the iris. The jet force should be minimal in this area to avoid damaging the zonules, which are often weak in patients with PEX. Washing and rinsing of the ICA and the entire anterior chamber is expected to facilitate evacuation of most of the macroscopic and microscopic PEX deposits.

A single or double tablet of a carbonic anhydrase inhibitor (acetazolamide 250 mg) was given to every patient about 3 and 6 h post-operatively to prevent post-operative IOP spikes after washout.

### Statistical analysis

Data in the study, unless otherwise specified, are presented as the mean value ± standard deviation (SD). Two-tailed Wilcoxon signed-rank test was used to evaluate statistical significance of 2 dependent data samples, that is pre- and post-surgery (matched pairs) values of visual acuity (VA) and IOP, for each corresponding time-point. Results were considered statistically significant for a *p* value equal or smaller than the significance level, i.e. 0.05.

## Results

In total, 190 eyes of 122 subjects, aged 73.8 ± 7.7 years, men (*N* = 36) and women (*N* = 86), underwent cataract surgery combined with Goniowash. The demographic data of the studied population are presented in the Table 1.

**Table 1:** Demographic synopsis of the study participants

Number of subjects	Number of eyes	Gender	Average age
122	190	Men *N* = 36 Women *N* = 86	73.8 ± 7.7 years

### Visual acuity

**Fig. 1:**
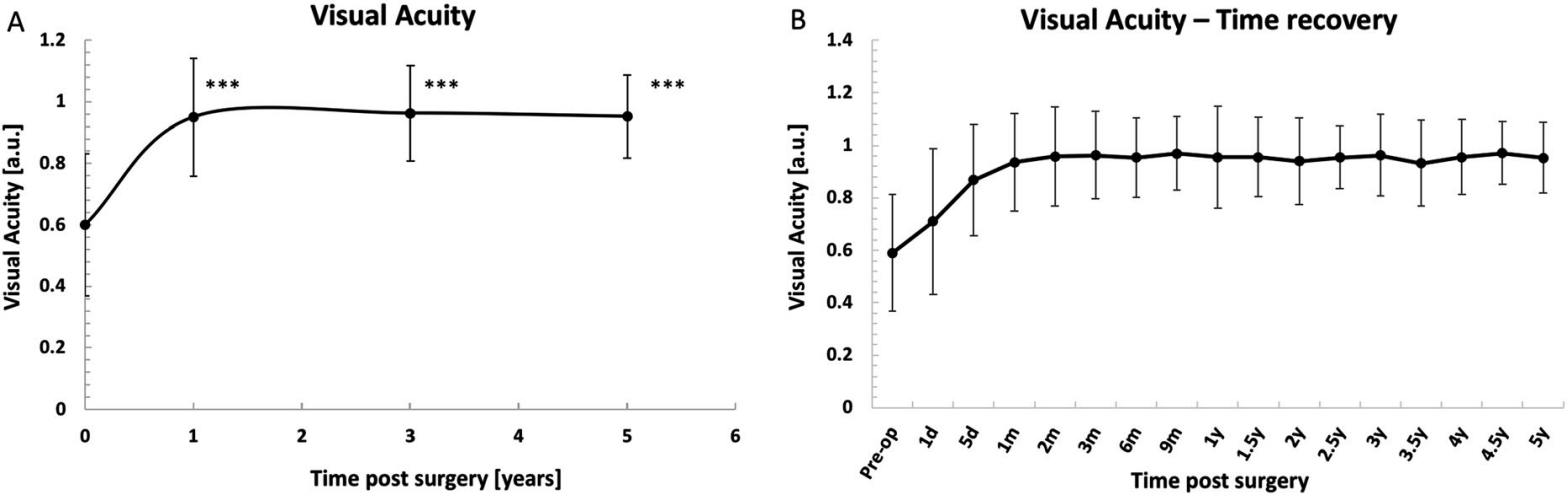
The effects of the cataract surgery with the use of Goniowash on the visual acuity. **A** Time effects of the surgical intervention on the Visual Acuity scores for 1 day, 1, 3 and 5 years follow-up respectively. **B** Long-term effects, up to 5 years, of the cataract surgery implementing Goniowash technique for each individual time-point of the follow-up. Statistics: Two-tailed Wilcoxon signed-rank test. *p < 0.05, **p < 0.1; ***p < 0.001

The statistical analysis compared pre- to post-operative VA scores of matched pairs. The results of analysis for 1 day, 1, 3, and 5 years are summarized in Table 2. At one day after surgery, a significant improvement in the VA was observed, i.e. from 0.6 ± 0.2 to 0.7 ± 0.3. VA was 1.0 ± 0.2, 1.0 ± 0.2, 1.0 ± 0.1 for year 1, 3 and 5, respectively. For all of time-points, the improvement from baseline was statistically significant (*p* < 0.001) (Fig. 1A). Figure 1B depicts the long-term effects (up to 5 years) of the cataract surgery implementing Goniowash technique on the visual acuity, for each individual time-point of the follow-up.

**Table 2:** Summary of the statistical analysis for the relevant time-points of the follow-up

Visual acuity	1 day	1 year	3 years	5 years
*N*	174	86	65	23
Pre-operative	0.6 ± 0.2	0.6 ± 0.2	0.6 ± 0.2	0.5 ± 0.2
Post-operative	0.7 ± 0.3	1.0 ± 0.2	1.0 ± 0.2	1.0 ± 0.1
*p* value	*p* < 0.001	*p* < 0.001	*p* < 0.001	*p* < 0.001
IOP_Liberal_				
*N*	186	92	61	23
Pre-operative [mmHg]	25.0 ± 7.0	26.4 ± 7.3	26.4 ± 7.4	28.3 ± 6.9
Post-operative [mmHg]	17.8 ± 7.7	15.9 ± 3.0	16.1 ± 3.3	16.8 ± 2.9
* p* value	*p* < 0.001	*p* < 0.001	*p* < 0.001	*p* < 0.001
IOP_Conservative_				
*N*	67	24	13	2
Pre-operative [mmHg]	22.9 ± 6.0	23.9 ± 6.7	25.1 ± 6.2	24.0 ± 0.0
Post-operative [mmHg]	16.6 ± 8.5	15.0 ± 3.3	16.3 ± 2.1	17.0 ± 5.7
* p* value	*p* < 0.001	*p* < 0.001	*p* < 0.001	*NA*

*IOP* intraocular pressure, *NA* not applicable

### Intraocular pressure

Since a significant part of the study patients were treated with IOP-lowering medications pre- and post-operatively, the effects of the surgical intervention on IOP was assessed using two distinct approaches. The so-called “liberal approach” evaluates the IOP values in all participants, regardless of the presence of treatment, whilst the “conservative approach” takes into account only those individuals that were not treated with any hypotensive before surgery or during the follow-up. (Table 2).

**Fig. 2:**
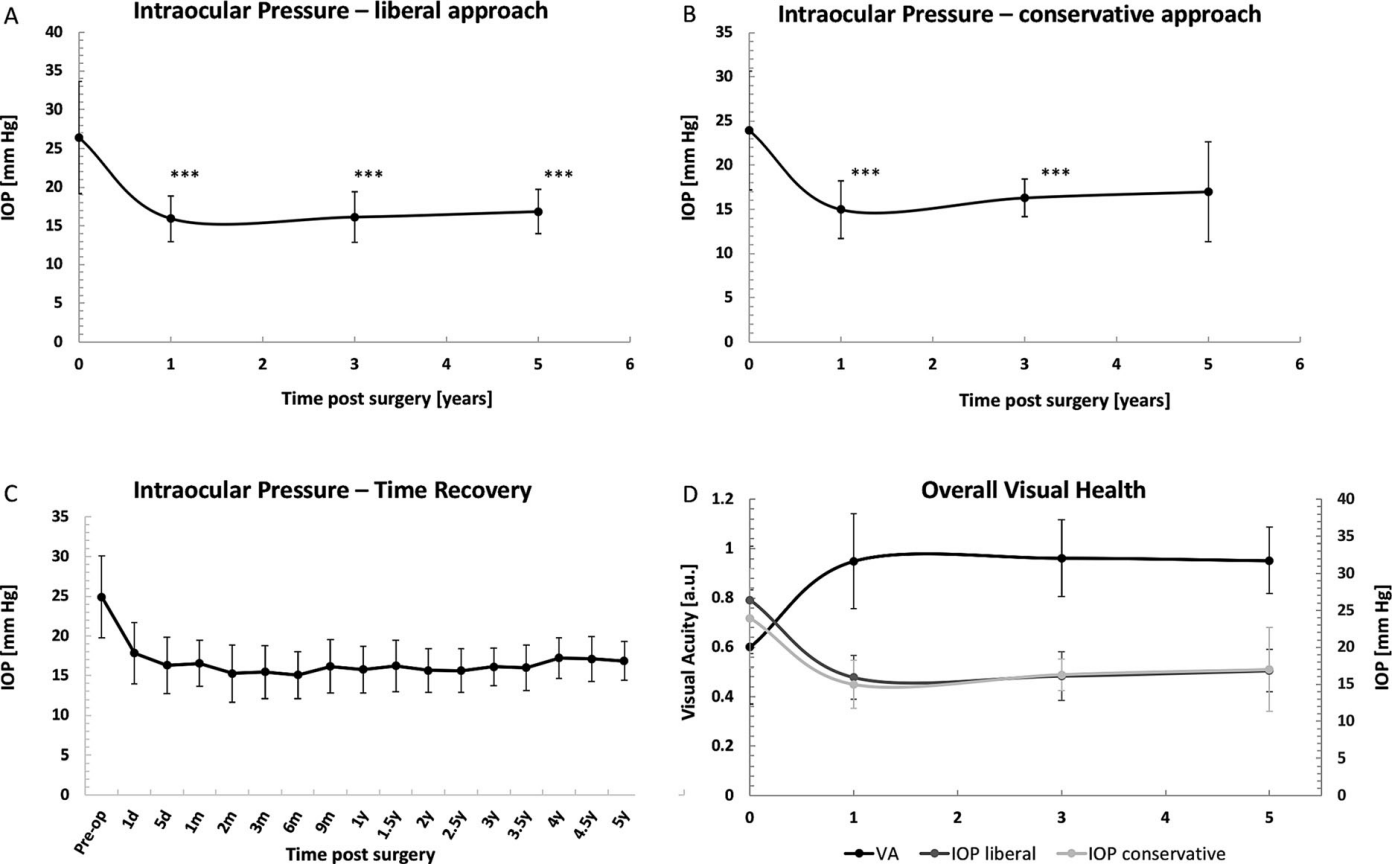
The effects of the cataract surgery with the use of Goniowash on the IOP. **A** Time effects of the surgical intervention on the IOP values in all patients, regardless of the use of IOP-decreasing treatment (“liberal” approach). The analysed time-points: 1 day, 1, 3 and 5 years of follow-up, respectively. **B** Time effects of the surgical intervention on the IOP values in patients that did not receive hypotensive drugs throughout the duration of the study (“conservative” approach). **C** Long-term effects, up to 5 years, of the cataract surgery implementing Goniowash technique for each individual time-point of the follow-up. **D** Comparison of the overall effects of the surgical intervention on the visual health parameters. Statistics: Two-tailed Wilcoxon signed-rank test. *p < 0.05, **p < 0.1; ***p < 0.001. Statistical significance could not have been assessed for the 5 years time-point in the “conservative” approach, due to low number of subjects

In the liberal approach, IOP values, assessed after 1 day post surgery, significantly decreased from 25.0 ± 6.9 to 17.8 ± 7.7 mmHg (29% decrease) (Table 2). The IOP values analyzed at later time-points continued to drop significantly. At 1 year post surgery, considerable decrease from the pre-operative baseline value of 26.4 ± 7.3 to 15.9 ± 3.0 mmHg (40% decrease) was observed. The detected decrease was stable at the further time-points, i.e. 39% at 3 years (decrease from 26.4 ± 7.4 to 16.1 ± 3.3 mmHg) and 41% at 5 years (decrease from 28.3 ± 7.0 to 16.8 ± 2.9 mmHg). For all of the discussed time-points, the effects were statistically significant (*p* < 0.001) (Fig. 2A).

In the conservative approach, the IOP values 1 day after surgery significantly decreased from 22.9 ± 6.2 to 16.6 ± 8.5 mmHg (Table 2). Furthermore, these values continued to further drop after 1 year post surgery, when a decrease from 23.9 ± 6.7 to 15.0 ± 3.3 mmHg, (37% decrease) was observed. This decrease was stable for later time-points, i.e. 35% at 3 years (decrease from 25.1 ± 6.2 to 16.3 ± 2.1 mmHg) and 30% at 5 years (decrease from 24.0 ± 0.0 to 17.0 ± 5.7 mmHg) (Fig. 2B).

Figure 2C depicts the global, time-dependent beneficial effects of the Goniowash technique on the IOP values, assessed using “liberal” approach. Figure 2D depicts superposition of the results for the liberal and conservative approaches.

Additional statistical test (two-sample, two-sided homoscedastic t-test) was utilized in order to assess if there is any statistical significance of the obtained IOP values between both approaches. The results of analysis, summarized in the Table 3, indicate no statistical significance for any of the analyzed time-points.

**Table 3:** Statistical comparison of the IOP values for the corresponding time-points of “liberal” and “conservative” relevant time-points of the follow-up

“Liberal” vs “Conservative” IOP values	Pre-op	1 year	3 years Pre-op	3 years	5 years Pre-op	5 years
*p* value	0.132	0.183	0.550	0.839	*NA*	*NA*

*IOP* intraocular pressure, *Pre-op* pre-operative, Statistics: Two-sample, two-sided homoscedastic t-test. *p < 0.05

### Medications

**Fig. 3:**
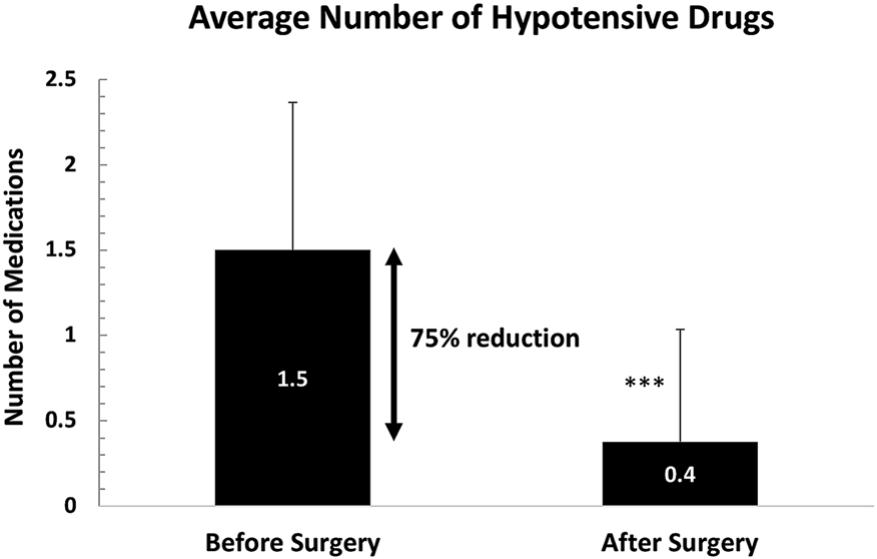
The effects of the cataract surgery with the use of Goniowash technique on the use of hypotensive drugs. Statistics: Two-tailed Wilcoxon signed-rank test. *p < 0.05, **p < 0.1; ***p < 0.001

The effects of the surgical intervention with the use of the Goniowash technique on the overall necessity to continue the hypotensive drug treatment were assessed. An overall reduction of 75% in the average number of hypotensive drugs applied to the study subjects, i.e. from 1.5 ± 0.8 before surgery to 0.4 ± 0.7 (*p* < 0.001), was observed as a consequence of the treatment using Goniowash (Fig. 3).

### Safety

No adverse events related to the surgical procedure were reported for any of the subjects.

## Discussion

The overall hypotensive effect of cataract surgery is assumed to be associated, amongst others with one or more of the following: widening of the anterior chamber angle; decrease in iridolenticular friction (linked to reduced liberation of exfoliation material and pigment granules after surgery); aspiration of deposited debris from the trabecular meshwork during surgery; partial removal of the exfoliation-producing anterior capsule; and improvement in trabecular outflow as a result of low-grade inflammation (mechanism is presumably the same as that seen in argon laser trabeculoplasties) [17]. The present study investigated the hypotensive effect of TM and ICA washout of PEX material using the newly designed Goniowash device. Our results show that the technique led to a significant (almost 40%) and sustained IOP decrease in combination with cataract surgery over several years of follow-up.

### IOP decrease

One of the effective surgical approaches utilized in the treatment of pseudoexfoliation glaucoma, pigment dispersion syndrome, and pigmentary glaucoma is the use of suction cannulas [19–21]. In various studies, Jacobi et al. discussed the benefit of clearing the area of the irido-corneal angle by the use of the trabecular aspiration. This technique is an invasive surgical procedure that can be combined with cataract surgery. More specifically, this method utilizes a cannula with pressure suction of about 100 to 200 mmHg, which is applied in the anterior chamber of the eye. It enables aspiration of the obstructive debris from the surface of the trabeculum [19, 20]. This surgical approach slightly differs from the Goniowash technique. Indeed, the principle of trabecular aspiration is based, as its name indicates, solely on the aspiration of the particles. Cleaning in Goniowash technique involves removing, dissolving and breaking of all particles before aspiration is applied, which facilitates the latter. According to the study of Jacobi et al. 1997 [20], morphological analysis of the results of trabecular aspiration clearly indicates the effectiveness of this technique in removing obstructive particles from the irido-corneal angle. As a consequence of the performed surgical intervention in the experimental setup, intraocular pressure decreased from 32.4 ± 7.2 mmHg before surgery to 18.4 ± 1.3 mmHg 2 years after treatment, which roughly corresponds to the 43% decrease in IOP values [19], an effect of similar magnitude to that observed in the current study using Goniowash technique. However, in another study of Jacobi performed on a group of 20 patients, the results were somewhat less convincing, with the IOP significantly decreased from 27 to 23.7 mmHg post surgery. The aspiration procedure was more efficient in the pigment dispersion syndrome patients. Most importantly, however, trabecular aspiration was unable to achieve long-term pressure control in either of the two groups, by contrary to what is observed in the current study.

In line with the outcome of the study at hand, significance of exfoliation material and pigment removal in the restoration of correct IOP values was confirmed in a 2-year prospective, multicenter cohort study, which evaluated the outcome of phacoemulsification procedure in cataract patients with (*n* = 71) and without exfoliation (*n* = 112) on the IOP values [13].

Amongst patients with exfoliation, 29 of them had XFG. In the study, the investigators observed that the IOP reduction was significantly greater in the XFS and XFG patients than both in control subjects without glaucoma and in patients with POAG. In addition, the investigators found that the hypotensive effect of the surgical approach in the exfoliation group correlated with the volume of irrigation fluid used. This observation prompts that the extent of exfoliation material and pigment removal may be a decisive factor in the rate of IOP decrease after cataract surgery. The results of the study are in line with the data from case series published previously [14, 15]. Finally, it is also noteworthy that the magnitude of post-operative IOP reduction seems to be proportional to the pre-operative IOP, i.e. the higher the pre-operative IOP value, the greater the hypotensive effect, which has been shown in a recent retrospective study on a large cohort of 1,122 XFS and XFG eyes followed for 7 years [16].

Regarding the use of the IOP-decreasing medications, the surgical approach with the use of Goniowash technique enabled 59% of concerned study subjects to completely stop glaucoma drugs. In comparison, 57% of subjects in the study of Jacobi et al. [19] withdrew from medications after 2 years post surgery. In addition, with the use of the Goniowash, an overall reduction of 75% in the average number of hypotensive drugs was observed.

### Visual acuity

Visual acuity scores were higher than the corresponding baseline values for all time-points during medical follow-up. In all cases, including the immediate effect after 1 day post surgery, these differences were statistically significant. In the study of Shoji and colleagues 2007 [22], patients who underwent a cataract surgery using phacoemulsification had a maximum increase in VA scores of 0.83 at 3 months, as compared to the 0.39 prior to surgery, which corresponds to the 112% improvement rate. In the current study, the highest VA score as compared to the pre-operative values was obtained after 5 years, i.e. from 0.5 ± 0.2 to 1.0 ± 0.1, (83% improvement). Of note is that for the corresponding time period, i.e. 3 months, an improvement of around 67% was recorded (data not shown). Even though these values seem to be somewhat lower than those seen in the study of Shoji [22], it is worth pointing out that baseline values of VA were also considerably higher in the current study than in the referenced study, i.e. 0.60 as compared to 0.39, as well as treated indications (glaucoma with pseudoexfoliation vs open angle glaucoma) differed, which might have had an overall impact on the final magnitude of improvement. A back-to-back comparison of both techniques used in the population of subjects suffering from the exact same medical indication would have to be performed in order to draw definite conclusions on the relative efficacy of both techniques.

Regarding the long-term effects on the VA scores, in the current study, the VA scores remained stable during the period of 5 years, indicating long-lasting improvement. The lack of VA scores decline was also observed in the investigation of Pfeiffer et al. 2015 [23] (up to 2 years) and Shoji et al. 2007 [22] (up to 3 years) who assessed the effects of the phacoemulsification procedure in the open angle glaucoma patients. In their study, Pfeiffer and colleagues reported that during first year of follow-up, 6% of patients that underwent standard cataract surgery, suffered from a loss of VA, which was higher than 2 lines. During the second year of medical follow-up, such decline was observed in 2% of patients. Shoji and colleagues on the other hand monitored the VA scores for the time period of 3 years. They observed an improvement in VA scores after 3 months post surgery, which remained stable throughout the follow-up period of 3 years. The current study provides evidence that when performing a cataract surgery using Goniowash technique, a clinically relevant, long-term improvement of VA scores up to 5 years may be expected.

### Safety

During the course of the study, no adverse events related to the functionality of the device were reported. Furthermore, no unexpected complications related to patients’ safety was observed. It is worth noting that 3 patients had to have IOP-decreasing medications prescribed post-operatively. In the publication of Rumelt et al. 2007 [24], the causes of accidental injuries related to the use of cannulas were evaluated, especially with regard to the case of accidental release of material by cannula during their use in the clinical theatre [24]. According to this study, an accidental release of material occurred in 0.88 ‰ of all cases, during the analyzed time period of 15 years (9 cases out of 10,230). Out of the 9 cases, 2 of them caused injuries of a retina, which had overall minor consequences on the patient’s visual acuity. The study concludes that accidental release of matter is a very rare event, and that in most cases the visual acuity of the patient is not affected [24].

According to the results presented above, irrigation cannulas appear to be devices with a good level of security and do not pose a health hazard to the patients.

### Study limitations

Although the data presented in the current study suggest that the use of the Goniowash technique is beneficial in subjects suffering from pseudoexfoliation syndrome, the study has some limitations. Due to the fact that the data were collected in a retrospective manner, without study control arm, it was not possible to adequately compare the obtained results with the patients of similar demographics (e.g. sex, age etc.), degree of PEX, baseline level of PEXS, or with those that underwent routine cataract surgery without Goniowash procedure. Nevertheless, when comparing the obtained results to the clinical evidence identified from available literature, in which no specific use of any irrigation or suction device is being mentioned, the obtained data on the achieved post-operative IOP decrease using Goniowash not only are more substantial (41% after 5 years, as compared to 14% after 12 months, reduced to 6% after 18 months [14], 25% after 3 months [25] and 28% after 6 months [26]), cover longer follow-up period (5 years as compared to 18 months [14]) but are also more robust in terms of sample size (*N* = 23 eyes at 5 years as compared to *N* = 6 eyes at 12 months and *N* = 4 eyes at 18 months [14]). It shall be pointed out, however, that the data collected within the scope of the study, especially pertaining to the long-term follow-up, may be partially subjected to bias, due to patients’ attrition.

## Conclusion

The data presented in the study substantiate efficacy and safety of the Goniowash technique used in combination with cataract surgery, in a large retrospective study format. Use of Goniowash technique in patients with pseudoexfoliation syndrome undergoing cataract surgery demonstrated a significant and sustained improvement in best-corrected visual acuity, as well as IOP decrease, and led to a reduction of hypotensive medications’ use. Importantly, this technique allows for avoiding incisions and sutures, thus respecting the anatomical structures of the eye without unnecessary invasive intervention [27]. Further work is required, however, to fully characterize the beneficial effects associated with the use of Goniowash technique, as compared to cataract surgery alone.

## References

[1] Streeten BW, Li ZY, Wallace RN, Eagle RC, Keshgegian AA (1992). Pseudoexfoliative fibrillopathy in visceral organs of a patient with pseudoexfoliation syndrome. Arch. Ophthalmol..

[2] Schlötzer-Schrehardt UM, Koca MR, Naumann GO, Volkholz H (1992). Pseudoexfoliation syndrome. Ocular manifestation of a systemic disorder?. Arch. Ophthalmol..

[3] Irkec M, Holló G, Konstas AGP (2012). Clinical features of exfoliative glaucoma. Exfoliation Syndrome and Exfoliative Glaucoma.

[4] Ritch R, Schlötzer-Schrehardt U (2001). Exfoliation SYNDROME. Surv. Ophthalmol..

[5] Ritch R (2007). Ultrasound biomicroscopic assessment of zonular appearance in exfoliation syndrome. Acta Ophthalmol Scand.

[6] Tetsumoto K, Schlötzer-Schrehardt U, Küchle M, Dörfler S, Naumann GO (1992). Precapsular layer of the anterior lens capsule in early pseudoexfoliation syndrome. Graefes Arch Clin Exp Ophthalmol.

[7] Schlötzer-Schrehardt U, Naumann GO (1995). Trabecular meshwork in pseudoexfoliation syndrome with and without open-angle glaucoma. A morphometric, ultrastructural study. Invest. Ophthalmol. Vis. Sci..

[8] Konstas AGP, Holló G, Ritch R, Schacknow PN, Samples RJ (2010). Exfoliative glaucoma. The Glaucoma Book. A Practical, Evidence-Based Approach to Patient Care.

[9] Thygesen J, Holló G, Konstas AGP (2012). Ocular clinical findings in exfoliation syndrome. Exfoliation Syndrome and Exfoliative Glaucoma.

[10] Tran VT (2009). UBM/slit-lamp-photo imaging of pseudoexfoliation deposits in the iridocorneal angle: imaging clues to the genesis of ocular hypertension. Int Ophthalmol.

[11] Tran VT (2015). Washout of pseudoexfoliation material combined with cataract surgery: a new surgical approach to lower intraocular pressure in pseudoexfoliation syndrome. Int Ophthalmol.

[12] Conway RM, Schlotzer-Schrehardt U, Kuchle M, Naumann GO (2004). Pseudoexfoliation syndrome: pathological manifestations of relevance to intraocular surgery. Clin Exp Ophthalmol.

[13] Damji KF, Konstas AGP, Liebmann JM (2006). Intraocular pressure following phacoemulsification in patients with and without exfoliation syndrome: a 2-year prospective study. Br J Ophthalmol.

[14] Merkur A, Damji KF, Mintsioulis G, Hodge WG (2001). Intraocular pressure decrease after phacoemulsification in patients with pseudoexfoliation syndrome. J Cataract Refract Surg.

[15] Shingleton BJ, Heltzer J, O’Donoghue MW (2003). Outcomes of phacoemulsification in patients with and without pseudoexfoliation syndrome. J Cataract Refract Surg.

[16] Shingleton BJ, Laul A, Nagao K (2008). Effect of phacoemulsification on intraocular pressure in eyes with pseudoexfoliation: single-surgeon series. J Cataract Refract Surg.

[17] Teus MA, de Benito-Llopis L, Holló G, Konstas AGP (2012). Update on cataract surgery in exfoliation syndrome. Exfoliation Syndrome and Exfoliative Glaucoma.

[18] Holló G, Katsanos A, Konstas AGP (2015). Management of exfoliative glaucoma: challenges and solutions. Clin Ophthalmol.

[19] Jacobi PC, Dietlein TS, Krieglstein GK (2000). Effect of trabecular aspiration on intraocular pressure in pigment dispersion syndrome and pigmentary glaucoma. Ophthalmology.

[20] Jacobi PC (1997). Antiglaucomatous trabecular surgery. Curr Opin Ophthalmol.

[21] Jacobi PC, Dietlein TS, Krieglstein GK (1998). Bimanual trabecular aspiration in pseudoexfoliation glaucoma. Ophthalmology.

[22] Shoji T (2007). Phacoviscocanalostomy versus cataract surgery only in patients with coexisting normal-tension glaucoma: midterm outcomes. J Cataract Refract Surg.

[23] Pfeiffer N (2015). A randomized trial of a Schlemm’s canal microstent with phacoemulsification for reducing intraocular pressure in open-angle glaucoma. Ophthalmology.

[24] Rumelt S (2007). The spectrum of iatrogenic intraocular injuries caused by inadvertent cannula release during anterior segment surgery. Arch Ophthalmol.

[25] Dosso A (1997). Exofoliation syndrome and phacoemulsification. J Cataract Refract Surg.

[26] Vahedian Z, Salmanroghani R, Fakhraie G, Moghimi S, Eslami Y, Zarei R, Mohammadi M (2015). Pseudoexfoliation syndrome: effect of phacoemulsification on intraocular pressure and its diurnal variation. J Curr Ophthalmol.

[27] Plateroti P, Plateroti AM, Abdolrahimzadeh S, Scuderi G (2015). Pseudoexfoliation syndrome and pseudoexfoliation glaucoma: a review of the literature with updates on surgical management. J Ophthalmol.

